# Visual evoked potentials in subgroups of migraine with aura patients

**DOI:** 10.1186/s10194-015-0577-6

**Published:** 2015-11-02

**Authors:** Gianluca Coppola, Martina Bracaglia, Davide Di Lenola, Cherubino Di Lorenzo, Mariano Serrao, Vincenzo Parisi, Antonio Di Renzo, Francesco Martelli, Antonello Fadda, Jean Schoenen, Francesco Pierelli

**Affiliations:** G.B. Bietti Foundation-IRCCS, Department of Neurophysiology of Vision and Neurophthalmology, Via Livenza 3, 00198 Rome, Italy; Department of Medical and Surgical Sciences and Biotechnologies, “Sapienza” University of Rome Polo Pontino, Latina, Italy; Fondazione Don Gnocchi, Milan, Italy; Istituto Superiore di Sanità, Dipartimento Tecnologie e Salute, Rome, Italy; Headache Research Unit, Department of Neurology-CHR Citadelle, University of Liège, Liège, Belgium; IRCCS-Neuromed, Pozzilli, IS Italy

**Keywords:** Migraine with aura, Visual aura, Complex aura, Visual evoked potentials, Habituation, Cortical excitability

## Abstract

**Background:**

Patients suffering from migraine with aura can have either pure visual auras or complex auras with sensory disturbances and dysphasia, or both. Few studies have searched for possible pathophysiological differences between these two subgroups of patients.

**Methods:**

Methods - Forty-seven migraine with aura patients were subdivided in a subgroup with exclusively visual auras (MA, *N =* 27) and another with complex neurological auras (MA+, *N =* 20). We recorded pattern-reversal visual evoked potentials (VEP: 15 min of arc cheques, 3.1 reversal per second, 600 sweeps) and measured amplitude and habituation (slope of the linear regression line of amplitude changes from the 1st to 6th block of 100 sweeps) for the N1-P1 and P1-N2 components in patients and, for comparison, in 30 healthy volunteers (HV) of similar age and gender distribution.

**Results:**

VEP N1-P1 habituation, i.e. amplitude decrement between 1st and 6th block, which was obvious in most HV (mean slope −0.50), was deficient in both MA (slope +0.01, *p =* 0.0001) and MA+ (−0.0049, *p =* 0.001) patients. However, VEP N1-P1 amplitudes across blocks were normal in MA patients, while they were significantly greater in MA+ patients than in HVs.

**Conclusions:**

Our findings suggest that in migraine with aura patients different aura phenotypes may be underpinned by different pathophysiological mechanisms. Pre-activation cortical excitability could be higher in patients with complex neurological auras than in those having pure visual auras or in healthy volunteers.

## Background

Migraine with aura (MA) is defined as attacks of neurological symptoms that last no more than 60 min and may be followed or accompanied by headache (International Classification of Headache Disorders 3beta 2013). The most common aura symptoms are visual (e.g. scintillating scotoma), while sensory and aphasic auras are present in a smaller proportion of patients [1, 2]. According to Rasmussen and Olesen [3], 51 % of migraine auras are purely visual, while 4 % comprise sensory symptoms in addition to the visual ones and 6 % language disturbances in addition to visual and sensory disturbances.

The most likely cause of the migraine aura, Leão’s cortical spreading depression (CSD), consists of a brief neuronal depolarisation followed by a long-lasting wave of neuronal depression that often spreads postero-anteriorly in the occipital cortex and can reach the parietal and/or temporal lobes [4, 5]. Indirect evidence for CSD occurrence in migraine patients stems from functional neuroimaging [6–8] and electrophysiological [9] studies. Although in animal models CSD is able to activate peripheral and central trigeminovascular neurons that underlie the migraine headache [10, 11], knowledge is lacking on the possible relation of CSD to interictal neural alterations that may predispose to migraine attacks.

During the last decade various research groups have demonstrated significant changes of bioelectrical activity in the visual cortex of migraine patients over the migraine cycle. In particular, cortical visual evoked potentials (VEPs) are used to infer the mass activity of visual cortical neurons. Most, though not all [12], VEP, recordings have shown that the brain of migraineurs with and without aura is characterized by an interictal deficit of habituation during stimulus repetition, and by its ictal normalization [13–16].

It was suggested that migraine with aura is a condition with a spectrum of clinical subtypes that likely differ in pathophysiological mechanisms [17]. Distinct electrophysiological abnormalities were especially found at the neuromuscular junction in patients suffering from complex neurological auras characterized by visual symptoms followed by sensorimotor and dysphasic symptoms [18] or from prolonged auras [19]. Using 1H-MR-spectroscopy, migraine patients with visual symptoms and at least one of paraesthesia, paresis, or dysphasia had a significant lactate increase in the visual cortex during sustained visual stimulation, while this was not the case in controls and patients with exclusive visual aura. In the latter group, however, lactate levels were already elevated at baseline and remained consistently high during the visual stimulation [20]. Besides its role as energy substrate of the brain, lactate acts as a neuromodulator and interacts with glutamate [21], GABA [22], and monoamines [23], which suggest that it is important in regulating the activity of cortical neurons. Lactate may increase to attenuate the electrical activity of excessively active neurons as observed in experimental models [22, 24, 25] and in healthy humans during sustained visual stimulation [26]. Considering these data and those obtained by NMR spectroscopy showing altered metabolic homeostasis of the migraineur’s brain [27, 28], it is of interest to verify whether activity of visual cortical neurons is increased in migraine patients suffering from complex auras respective to those experiencing pure visual symptoms. We decided therefore to compare amplitude and habituation of pattern reversal VEP in healthy volunteers, migraine patients with pure visual auras, and in patients with complex neurological auras including at least one of sensory and language symptoms in addition to visual disturbances. Considering the abovementioned NMR spectroscopy studies and our prior interictal VEP studies in migraine with aura [14, 15], we reasoned that subgroups of migraine with aura patients would show both common and specific neurophysiological abnormalities. We hypothesized that VEP amplitude would be higher in migraine with complex aura than in migraine with pure visual aura, while habituation would be equally deficient in both MA subgroups.

## Methods

### Subjects

We initially enrolled 58 consecutive migraine patients with typical aura (MAtot, ICHD-3beta code 1.2.1.1) who attended our headache clinic. We discarded recordings of 10 patients who did not fulfil our primary inclusion criteria (see below), and of one patient because he was an outlier. The final analysis set comprises therefore 47 patients (32 women, mean age 31.8 years). Patients were subdivided into those who experienced pure visual aura (MA, *N =* 27) and those who had in addition paraesthesia and/or dysphasia (i.e. complex neurological auras; MA+, *N =* 20). Auras usually developed gradually and were followed by headache. None had hemiplegic or brainstem auras or persistent aura without infarction. All patients had a varying combination of attacks with or without aura. We took information on various clinical characteristics by collecting up to two-month headache diaries at the time of the screening visit and the day of the recording session. Patients had to indicate duration of migraine history (years), attack frequency (n/month), attack duration (hours), and number of days elapsed since the last migraine attack (in 35 out of 47 patients) (Table 1).

**Table 1:** Clinical and demographic characteristics of healthy volunteers (HV), the total group of migraine with aura patients (MAtot) and its subgroups with pure visual aura (MA) or visual aura associated with paraesthesia and/or dysphasia (MA+). Data are expressed as means ± SD

Characteristics	HV (*n =* 30)	MAtot (*n =* 47)	MA (*n =* 27)	MA+ (*n =* 20)
Women (*n*)	18	32	17	15
Age (years)	33.4 ± 13.4	31.8 ± 9.3	31.7 ± 9.4	32.5 ± 9.5
Duration of migraine history (years)		16.1 ± 9.9	15.3 ± 9.5	17.0 ± 10.5
Attack frequency/month (*n*)		2.5 ± 2.6	2.1 ± 2.2	2.9 ± 3.0
Attack duration (hours)		28.8 ± 25.4	29.9 ± 27.2	27.4 ± 23.3
Days since the last migraine attack		17.5 ± 16.1	15.3 ± 16.4	20.6 ± 15.9

A primary inclusion criterion was being attack-free for at least 3 days before and after the recording sessions, as checked by collecting headache diaries, and by telephone or e-mail interviews. Migraine patients were recorded in the interictal period. The time range of 3 days was chosen to avoid the accidental inclusion of patients recorded during an attack. In fact, according to the International classification of Headache Disorders, a migraine attack can last up to 3 days. Of the patients initially recorded, ten patients had an attack within 3 days after the recording session, and thus their VEP data were not included in the present analysis. We chose to focus on interictal recordings because some previous studies showed that habituation reflects the periodicity between 2 migraine attacks, i.e. is lacking between attacks and normalizes immediately before and during an attack [15, 29]. For comparison, we enrolled a group of 30 age-matched healthy volunteers (18 women, mean age 33.4 years) recruited among medical school students and healthcare professionals, randomly recorded between patients. Exclusion criteria were regular medication intake (i.e. antibiotics, corticosteroids, antidepressants, benzodiazepines, prophylactic migraine drugs) except for the contraceptive pill, failure to reach a best-corrected visual acuity of > 8/10, history of other neurological diseases, systemic hypertension, diabetes or other metabolic disorders, connective or autoimmune diseases, and any other type of primary or secondary headache. Female participants were always recorded at mid-cycle. All participants (HV and MwA) were naïve to the study procedure. We did not give any recommendation and/or information to HV or patients about potential clinical benefits or harms associated with the recordings.

All participants received a complete description of the study and granted written informed consent. The ethical review board “Sapienza” University of Rome, Polo Pontino, approved the project.

### Visual evoked potentials

Subjects were sitting in an acoustically isolated room with dimmed lights in front of a TV monitor surrounded by a uniform luminance field of 5 cd/m^2^. To obtain a stable pupillary diameter, each subject adapted to the ambient room light for 10 min before the VEP recordings. VEP were elicited by right monocular stimulation. Visual stimuli consisted of full-field checkerboard patterns (contrast 80 %, mean luminance 50 cd/m^2^) generated on a TV monitor; the reversal rate was 1.55 Hz (3.1 reversal per second)). At the viewing distance of 114 cm, the single checks subtended a visual angle of 15 min, while the checkerboard subtended 23°. Recordings were done with the best corrected visual acuity of > 8/10 at the viewing distance. Subjects were instructed to fixate with their right eye a red dot in the middle of the screen with the contralateral eye covered by a patch to maintain stable fixation. VEP were recorded from the scalp through silver cup electrodes positioned at Oz (active electrode) and at Fz (reference electrode, 10/20 system). A ground electrode was placed on the right forearm. Signals were amplified by Digitimer™ D360 pre-amplifiers (band-pass 0.05–2000 Hz, gain 1000) and recorded with a CED™ power 1401 device (Cambridge Electronic Design Ltd, Cambridge, UK). A total of 600 consecutive sweeps each lasting 200 ms were collected and sampled at 4000 Hz.

After applying off-line a 35Hz low-pass digital filter, cortical responses were partitioned in 6 sequential blocks of 100, consisting of at least 95 artifact-free sweeps. Responses in each block were averaged off-line (“block averages”) using the Signal™ software package version 4.10 (CED Ltd). Artefacts were automatically rejected using the Signal™ artefact rejection tool if the signal amplitude exceeded 90 % of analog-to-digital converter (ADC) range and was controlled by visual inspection. Through this approach, we made sure to exclude all severe artefacts but not to remove any signal systematically because background EEG amplitudes vary between subjects. The EP-signal was corrected off-line for DC-drifts, eye movements and blinks.

**Fig. 1:**
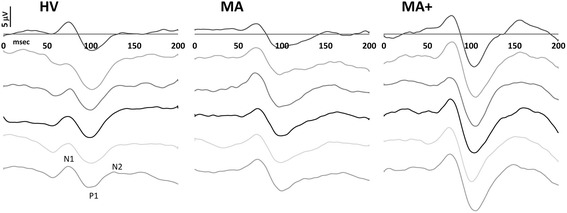
Representative recordings (low pass filter 35 Hz) of visual evoked potentials in a healthy volunteer (HV), a migraine patient with pure visual aura (MA), and a migraine patient with complex aura (MA+) recorded between attacks. The 6 successive blocks of 100 averaged responses from top to bottom illustrate the difference between the 3 subjects in 1st block N1-P1 and P1-N2 amplitudes, and in amplitude change (habituation) over the 6 blocks

VEP components were identified according to their latencies: N1 was defined as the most negative peak between 60 and 90 ms, P1 as the most positive peak following N1 between 80 and 120 ms and N2 as the most negative peak following P1 at between 125 and 150 ms (Fig. 1). We measured the peak-to-peak amplitude of the N1–P1 and P1–N2 complexes. Habituation was defined as the slope of the linear regression line for the 6 blocks. All recordings were collected in the morning (between 09.00 and 11.00 a.m.) by the same investigators (D.D.L and C.D.L.), who did not meet the participants prior to the examination, since they were not involved in recruitment and inclusion of subjects. All recordings were numbered anonymously and analyzed blindly off-line by one investigator (M.B.), who was not blinded to the order of the blocks.

### Statistical analysis

We used the Statistica for Windows (StatSoft Inc.), version 8.0 for all analyses. Preliminary descriptive analysis showed that the VEP N1–P1 and P1–N2 peak-to-peak amplitudes of the six blocks and the habituation slopes had a non-normal distribution. After log transformation, all data reached normal distribution (Kolmogorov-Smirnov test, *p >* 0.2).

A General Linear Model approach was used to analyze the “between-factor” × “within-factors” interaction effect. The between-subject factor was “group” (HV *vs*. MAtot or HV *vs.* MA subgroups); the within-subject factor was “blocks”. Two models of repeated measures ANOVA (rm-ANOVA) followed by univariate ANOVAs were employed to investigate the interaction effect. Univariate results were analyzed only if Wilks’ Lambda multivariate significance criterion was achieved. The sphericity of the covariance matrix was verified with the Mauchly Sphericity Test; in the case of violation of the sphericity assumption, Greenhouse-Geisser (G-G) epsilon (*ε*) adjustment was used. In rm-ANOVA and ANOVA models, partial eta^2^$\left({\eta}_p^2\right)$ and observed power (op) were used as measures of effect size and power, respectively. To define which comparison(s) contributed to the major effects, post hoc tests were performed with Tukey Honest Significant Difference (HSD) test.

A regression analysis was used to disclose linear trends in VEP amplitude across blocks (slope) in each group. For slope, we employed ANOVA with group factor “group” (HV *vs*. MAtot or HV *vs.* MA subgroups), using Tukey test for post hoc analysis. Also for ANOVA partial eta^2^ and op was used. Statistical significance was set at *p <* 0.05.

Pearson’s correlation test was used to search for correlations among VEP amplitude slopes and clinical variables (duration of migraine history, attack frequency, attack duration, days since the last migraine attack).

## Results

Recordings from 47 participants who fulfilled the inclusion criteria yielded analysable VEP data. The two patient subgroups MA and MA+ did not differ in other clinical features (Table 1).

### Total group of migraine with aura patients (MAtot)

N1, P1 and N2 latencies were not significantly different between HV and MAtot (*p >* 0.05) (Table 2).

**Table 2:** Latencies (in milliseconds) of VEPs in healthy volunteers (HV), the total group of migraine with aura patients (MAtot) and its subgroups with pure visual aura (MA) or visual aura associated with paraesthesia and/or dysphasia (MA+). Data are expressed as means ± SD.

Electrophysiological parameters (ms)	HV (*n =* 30)	MAtot (*n =* 47)	MA (*n =* 27)	MA+ (*n =* 20)
N1 (N75)	76.5 ± 6.4	75.7 ± 5.2	75.4 ± 5.8	75.9 ± 4.6
P1 (P100)	103.5 ± 5.9	103.1 ± 6.7	102.9 ± 5.7	103.4 ± 8.0
N2 (145)	144.2 ± 10.4	141.7 ± 11.9	140.6 ± 9.6	142.6 ± 11.4

**Table 3:** N1–P1 VEP component amplitude (μV) and habituation slope in healthy volunteers (HV), the total group of migraine with aura patients (MAtot) and its subgroups with pure visual aura (MA) or visual aura associated with paraesthesia and/or dysphasia (MA+). Data are expressed as means ± SD

N1-P1	HV (*n =* 30)	MAtot (*n =* 47)	MA (*n =* 27)	MA+ (*n =* 20)
1^st^ amplitude block (μV)	6.97 ± 2.90	7.28 ± 3.23	6.53 ± 3.36	8.27 ± 2.83
2^nd^ amplitude block (μV)	7.15 ± 3.02	7.39 ± 3.23	6.43 ± 3.29	8.69 ± 2.70
3^rd^ amplitude block (μV)	6.87 ± 2.79	7.40 ± 2.96	6.49 ± 2.83	8.64 ± 2.74
4^th^ amplitude block (μV)	6.55 ± 2.74	7.16 ± 3.17	6.12 ± 3.07	8.57 ± 2.81
5^th^ amplitude block (μV)	6.25 ± 2.57	7.34 ± 3.00	6.49 ± 2.97	8.49 ± 2.70
6^th^ amplitude block (μV)	5.97 ± 2.63	7.42 ± 3.02	6.65 ± 3.09	8.45 ± 2.64
Slope	−0.50 ± 0.36	+0.006 ± 0.40	+0.01 ± 0.30	+0.0049 ± 0.18

**Table 4:** P1-N2 VEP component amplitude (μV) and habituation slope in healthy volunteers (HV), the total group of migraine with aura patients (MAtot) and its subgroups with pure visual aura (MA) or visual aura associated with paraesthesia and/or dysphasia (MA+). Data are expressed as means ± SD

P1-N2	HV (*n =* 30)	MAtot (*n =* 47)	MA (*n =* 27)	MA+ (*n =* 20)
1^st^ amplitude block (μV)	6.59 ± 3.16	7.00 ± 3.07	6.12 ± 2.65	8.18 ± 3.26
2^nd^ amplitude block (μV)	6.49 ± 3.03	6.87 ± 3.26	5.88 ± 2.55	8.20 ± 3.70
3^rd^ amplitude block (μV)	6.49 ± 2.94	6.62 ± 3.10	5.67 ± 2.66	7.91 ± 3.25
4^th^ amplitude block (μV)	5.99 ± 2.85	6.32 ± 2.88	5.37 ± 2.36	7.60 ± 3.08
5^th^ amplitude block (μV)	6.26 ± 2.59	6.84 ± 2.93	6.01 ± 2.45	7.96 ± 3.19
6^th^ amplitude block (μV)	5.61 ± 2.86	6.50 ± 2.78	5.78 ± 2.70	7.47 ± 2.64
Slope	−0.35 ± 0.73	−0.09 ± 0.42	−0.06 ± 0.47	−0.13 ± 0.35

**Fig. 2:**
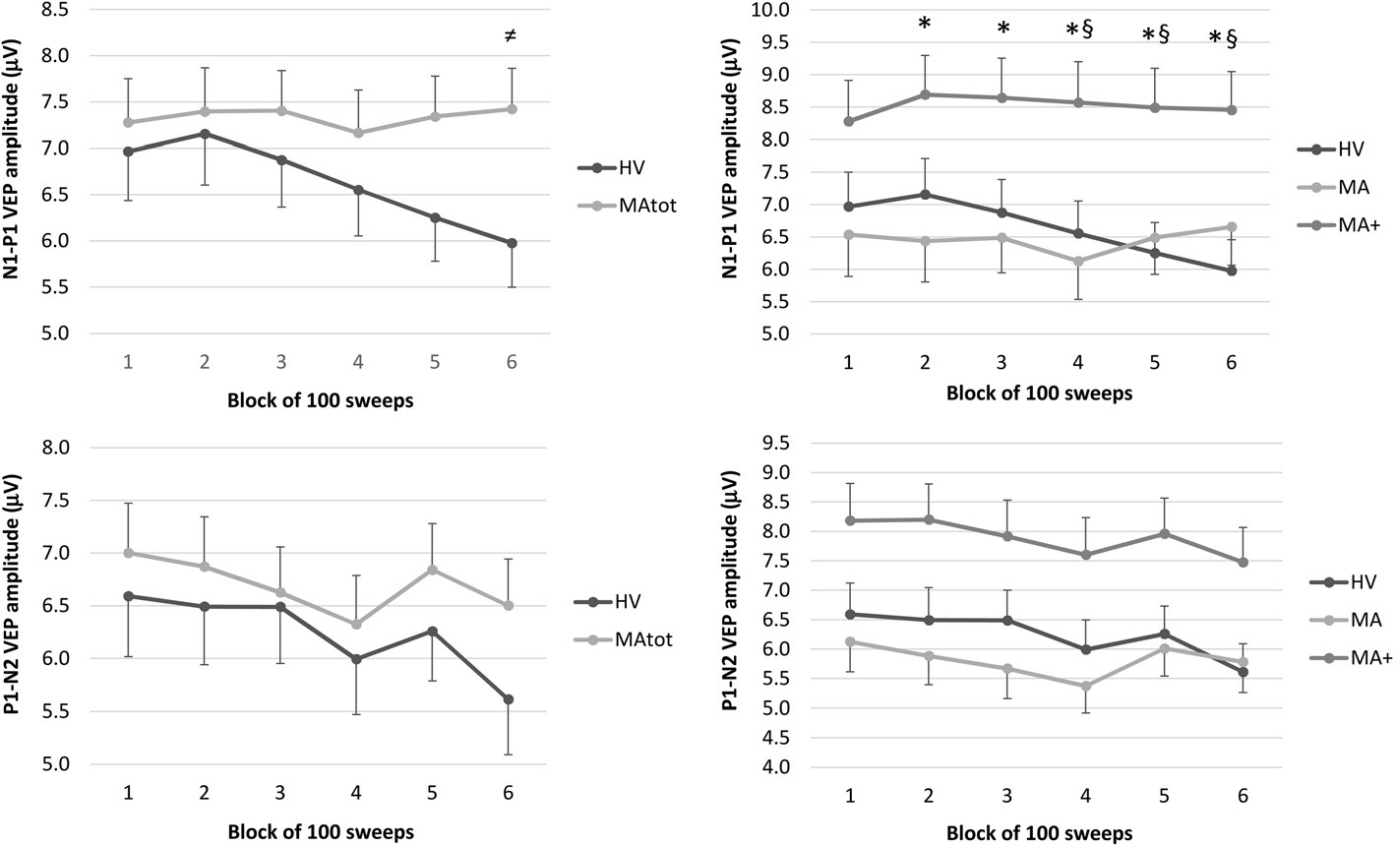
Raw amplitudes (mean ± SEM) of N1-P1 (upper graphs) and P1-N2 (lower graphs) VEP components in 6 sequential blocks of 100 recordings. On the left healthy volunteers [HV, *n =* 30] are compared to the total group of migraine with aura patients [MAtot, *n =* 47]; on the right they are compared to the 2 subgroups of patients with pure visual aura [MA, *n =* 27] and patients with complex aura [MA+, *n =* 20]. ≠ *p <* 0.05 MAtot vs HV; *p < 0.05 MA+ vs MA; § *p <* 0.05 MA+ vs HV

In the rm-ANOVA model with N1–P1 peak-peak amplitude as dependent variable, multivariate test was significant for the “*group*” × “*blocks*” interaction effect (Wilks’ Lambda = 0.745, F_5,71_ = 4.862, *p =* 0.0007) (Table 3). After checking that the sphericity assumption was not violated (Mauchley Test: *p =* 0.104), univariate rm-ANOVAs for N1–P1 peak-peak amplitude confirmed the significant interaction factor effect (F_5,375_ = 5.261, *p =* 0.0001, partial η^2^ = 0.066, op = 0.988) observed (see above) at the multivariate test. Post-hoc analysis showed that VEP amplitudes differed between groups only in the last block (5.97 μV in HV vs. 7.42 μV in MAtot, *p =* 0.038, raw data are shown in Fig. 2). In HV, N1–P1 amplitude was significantly lower in the 6th compared to the 1st block (*p =* 0.0008). This was not so in MAtot, where this comparison did not reach the significance level (*p =* 0.994).

In the rm-ANOVA model with P1-N2 peak-peak amplitude as dependent variable, multivariate test was not significant for the “*group*” × “*blocks*” interaction effect (Wilks’ Lambda = 0.869, F_5,71_ = 2.149, *p =* 0.069) (Table 4).

**Fig. 3:**
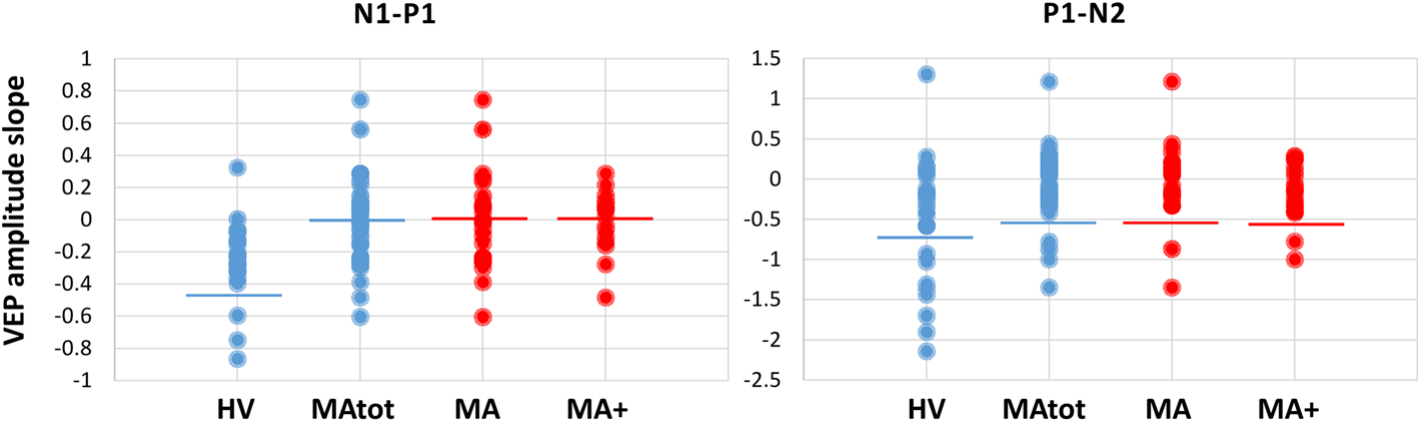
Raw habituation slope of VEP N1-P1 and P1–N2 peak-to-peak amplitudes (mean ± SEM) over 6 sequential blocks of 100 averaged responses in healthy volunteers (HV, *n =* 30), patients with pure visual aura (MA, *n =* 27), patients with complex aura (MA+, *n =* 20) and the 2 latter groups combined (MAtot, *n =* 47)

The linear regression N1–P1 slope of VEP amplitudes over all blocks differed significantly between the two groups (F_1,75_ = 24.493, *p <* 0.0001, partial η^2^ = 0.246, op = 0.998; raw data are shown in Fig. 3). The P1–N2 slope of the linear regression analysis was not different between groups (F_1,75_ = 3.312, *p =* 0.073, partial η^2^ = 0.042, op = 0.435; Fig. 3).

In the MAtot group the N1–P1 amplitude slope correlated positively with the number of days elapsed since the last migraine attack (r = 0.351, *p =* 0.045). There were no other significant correlation between neurophysiological and clinical data.

### Subgroups of migraine with aura patients

N1, P1 and N2 latencies were not significantly different between HV, MA or MA+ (*P >* 0.05) (Table 2).

In the rm-ANOVA model with N1–P1 peak-peak amplitude as dependent variable, multivariate test was significant for the “group” × “blocks” interaction effect (Wilks’ Lambda = 0.711, F_10,140_ = 2.608, *p =* 0.006). After checking that the sphericity assumption was not violated (Mauchley Test: *p =* 0.126), univariate rm-ANOVAs for N1-P1 peak-peak amplitude confirmed the significant interaction factor effect (F_10,370_ = 3.025, *p =* 0.001, partial η^2^ = 0.076, op = 0.982) observed (see above) at the multivariate test. On post-hoc analysis there was a significant increase of N1-P1 VEP amplitude from the 2^nd^ to the 6^th^ block in MA+ compared with MA, and from the 4^th^ to the 6th block in MA+ compared with HV (row data are shown in Fig. 2). In both MA and MA+, the comparison between the 6th and the 1st N1-P1 amplitude block did not reach the significance level (*p >* 0.05).

In the rm-ANOVA model with P1–N2 peak-peak amplitude as dependent variable, multivariate test was not significant for the “group” × “blocks” interaction effect (Wilks’ Lambda = 0.834, F_10,140_ = 1.335, *p =* 0.218).

The linear regression N1–P1 slope of VEP amplitudes over all blocks differed significantly between the three groups (F_2,74_ = 12.219, *p <* 0.0001, partial η^2^ = 0.248, op = 0.995; raw data are shown in Fig. 2). Post-hoc analysis showed that the slope of N1–P1 VEP amplitude changes over all 6 blocks was less steep in MA and in MA+ patients than in HV (*p =* 0.0001, *p =* 0.001 respectively, raw data are shown in Fig. 3), but it was equally steep between MA subgroups (*p =* 0.894).

The P1–N2 slope of the linear regression analysis was not different between groups (F_2,74_ = 1.720, *p =* 0.186, partial η^2^ = 0.044, op = 0.351; Fig. 3).

**Fig. 4:**
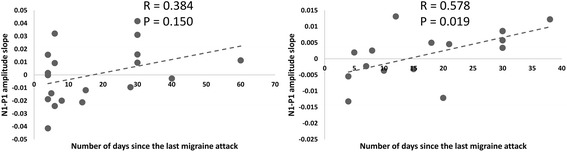
Correlation between the days elapsed between the recordings and the last migraine attack and the slope of N1–P1 VEP amplitude changes over 6 sequential blocks of averaged responses (linear regression: dashed line). This correlation was significant in the group of patients with complex aura (MA+, right panel), but not in patients with pure visual aura (MA, left panel)

In MA+, Pearson’s test disclosed that the N1–P1 habituation slope correlated negatively with attack frequency (r = −0.489, *p =* 0.034) and positively with days elapsed since last attack (r = 0.578, *p =* 0.019), correlations that were not found in MA patients (Fig. 4).

## Discussion

The purpose of the present study was to extend our electrophysiological investigations of visual cortex reactivity in migraine by searching for differences between two distinct phenotypes of migraine with aura.

First, we confirm the previous finding that during continuous stimulation amplitude of the VEP N1–P1 component, but not of P1–N2, does not habituate over sequential blocks of averaged responses in migraine with aura patients between attacks while it does so in healthy volunteers [15]. An additional novel finding is that, relative to HV, VEP N1–P1 habituation is deficient both in migraine with pure visual aura (MA) and in patients with complex aura (MA+).

A second striking result is that the amplitude of visual responses differs between patients having pure visual aura and those with complex auras. MA+ patients consistently have greater N1–P1 VEP amplitudes than MA patients. Contrary to MA+, MA patients do not differ from healthy volunteers in VEP N1–P1 and P1–N2 block amplitudes, although they have reduced habituation over the 6 sequential blocks of 100 averaged VEP responses.

To the best of our knowledge, this is the first study of visual evoked responses in patients with different migraine aura phenotypes. It identifies within the migraine spectrum a subgroup of patients with complex neurological auras in whom excitability of the visual cortex appears genuinely increased, as evidenced by an increased VEP N1–P1 amplitude and decreased habituation. Previous VEP studies have yielded conflicting results in groups of migraine with aura (MwA) patients without phenotype distinction. In some reports the grand-average of VEP N1–P1 and/or P1–N2 amplitudes was found greater in MwA patients than in controls [30–35] and/or in migraine without aura (MO) patients [31, 36, 37]. The amplitude of steady-state VEP harmonics was also larger in MwA than in MO or HV [38]. In other studies, on the contrary, VEP amplitudes were found reduced in MwA [39], even when compared to MO [40]. Most often, VEP amplitudes in MwA were reported to be in the normal range [13–15, 41–44]. Our finding of low or normal visual cortex excitability in patients with pure visual auras, which is similar to migraine without aura patients [45] but contrasts with increased VEP amplitude and deficient habituation in patients with complex auras, may help to explain some of the abovementioned discrepant results. In fact, pooling patients with different migraine phenotypes (MO and MwA or different MA subgroups) in different proportions increases the variance of VEP results between studies. This probably fueled in part the controversy about the presence or not of interictal cortical hyperexcitability in migraine. In a previous review paper, we reasoned that in its strict physiological definition of a stimulus–response curve, the cortex would be hyperexcitable if it generates a response to a subliminal stimulus and/or if its response to a supraliminal stimulus is increased in amplitude. Because in most previous studies VEP amplitude in MO patients and, according to the present results, also in MA patients, increases during stimulus repetition, while remaining within a normal range (see Fig. 2), we proposed to abandon the general term “hyperexcitability” in favour of “hyperresponsivity” to characterise the response pattern of the migrainous brain to repeated stimulations [46]. As shown here, the functional abnormality is clearly different in MA+ patients in whom the initial VEP amplitude to a low number of stimuli is increased compared to both HV and MA, indicating that their visual cortex is genuinely hyperexcitable.

From a pathophysiological point of view, it is interesting to compare MA+ and chronic migraine that is also thought to be associated with true cortical hyperexcitability. The evidence in chronic migraine comes from studies of somatosensory evoked potentials [47] and magnetoencephalographic visual evoked responses [48]. The difference with MA+ is that in the latter VEP amplitude was increased in virtually all blocks of averagings and habituation was deficient over 6 blocks, while in chronic migraine only the 1st block of averaged visual or somatosensory responses was increased in amplitude, but not the subsequent blocks, leaving habituation normal. The electrophysiological pattern in migraine with complex neurological auras may therefore suggest that the visual cortex is locked in a state of persistent hyperexcitability.

The pathophysiological determinants of different aura phenotypes and related differences in interictal visual evoked potential profiles remain speculative. Cortical spreading depression (CSD) is thought to be the pathophysiological substrate of the migraine aura. CSD is an electrochemical wave that usually starts in the posterior regions of the brain and spreads anteriorly at approx. 3 mm/min, accompanied by biphasic cerebral blood flow changes [4]. In several brain imaging studies performed during attacks, though not in all, [49, 50] the vascular and metabolic changes accompanying the migraine aura spread more anteriorly in patients with complex neurological symptoms and hemiplegia than in those with only visual disturbances. The recovery from CSD depends largely on intact neurovascular coupling to match the increased energy demand and to restore ion gradients via the Na^+^/K^+^ ATPase pump [51]. The distance, to which CSD spreads during MwA attacks, and thus the clinical phenotype of the aura, depends on the balance between factors that predispose the brain to CSD and others that inhibit CSD and allow the parenchyma to recover.

The neurovascular tone is modulated by local factors such as oxygen availability or lactate concentrations, and by subcortical monoaminergic projections [52, 53]. During continuous visual stimulation neurovascular coupling is impaired in migraine patients between attacks, especially in migraine with aura [34, 54, 55]. There is also circumstantial evidence from biochemical and functional neuroimaging studies that monoaminergic, in particular serotonergic, transmission from the brainstem to the thalamus and cortex is altered in migraine [56]. Finally, convergent data from various laboratories have shown that the mitochondrial energy reserve and ATP levels are significantly reduced in the brain of migraineurs between attacks [27, 28]. Based on these biochemical and functional data, we have proposed that migraine is characterized interictally by a cycling dysregulation of the serotoninergic control of thalamo-cortical activity that causes varying degrees of cortical hyperresponsivity and thus increased energy demands, which, under the influence of triggering or aggravating factors, may disrupt homeostasis and lead to an attack [14, 57].

Several studies suggest that the abnormalities of energy metabolism could be more pronounced in migraine with complex neurological aura. The phosphocreatine/phosphate (PCr/Pi) ratio, a marker of the brain’s energy reserve, differed significantly between patients with different aura phenotypes and was lowest in those with more complex auras [58]. In a 1H-MR-spectroscopy study [20] MA+ patients had a significant increase of lactate in the visual cortex during sustained visual stimulation, while this was not the case in HV and MA patients. Variants in the mitochondrial DNA, such as those that distinguish responders from non-responders to preventive anti-migraine treatment with riboflavin [59], could play a role in the metabolic differences between aura phenotypes.

That genetic load can influence CSD patterns and severity is evidenced by the studies of the “knock-in” mice wearing CACNA1A [60] or ATP1A2 [61] mutations found respectively in familial hemiplegic migraine (FHM) type 1 and 2. In FHM1 mice having the S218L mutation that causes a more severe clinical phenotype in patients, CSD are more frequent and more spread out (up to the striatum) than in mice with the R192Q mutation. As mentioned, the common form of migraine with aura is not associated with the mutations found in FHM, but merely with common variants in a number of loci identified on genome-wide association studies (GWAS) that are seemingly not much different from those found in migraine without aura [62]. It remains to be determined whether the combination of such common genetic variants and their association with mitochondrial DNA variants may influence the clinical migraine phenotype, including that of the aura.

One can only speculate on the possible relation between the ictal phenomena, i.e. CSD and its spreading, and the VEP abnormalities found interictally. We know of only one study in photosensitive subjects with a photo-paroxysmal response to intermittent photic stimulation where increased VEP amplitude was correlated with spread of the paroxysmal EEG activity to more anterior brain areas [63]. In photo-paroxysmal responses and photically induced seizures, this could be the electrophysiological correlate of increased functional connectivity between occipital and parieto-temporo-frontal networks under the control of the thalamus [64–66]. A recent study showing in animals that CSD can activate the thalamic reticular nuclei that controls the flow of sensory information to the cortex, is therefore of major interest [67]. Translated to migraine pathophysiology, one may hypothesize that repeated thalamic activation by CSD could worsen the interictal impairment of thalamic/thalamocortical activity in migraine with complex auras [14, 68–71]. Studies correlating aura frequency and duration of the disorder with thalamic/thalamocortical activity in MwA are necessary to test this hypothesis.

Whatever the possible relation between ictal CSD and interictal VEP might be, the pathophysiological mechanisms underlying VEP habituation are not permanently influenced by the ictal phenomena, even in MA+ patients. In the latter, indeed, like in MwA patients overall and in migraine without aura [15], the VEP habituation deficit is obvious between attacks. Moreover, in MA+, but not in MA patients, it worsens progressively with time elapsed since the last migraine attack and decreases with increased attack frequency; in other words, VEP habituation increases with proximity to an attack. To explain this difference between MA+ and MA, we speculate that MA+ patients are carrying the most pronounced genetic load predisposing them to more prominent pathophysiological dysfunctions. For instance, we intend to explore the possibility that MA+ is the migraine with aura phenotype with the most pronounced deficit of short-range lateral inhibition within the visual cortex, an abnormality that we also found directly related to the distance from the last attack in a previous study of a mixed group of migraine with and without aura patients [15]. Taken together with our present results, this would indicate that the inhibitory performance and habituation with stimulus repetition decreases with the distance from the last migraine attack. A psychophysical study using visual metacontrast masking test, found a similar correlation between inhibitory processes and the number of days elapsed since the last attack [72]. The biochemical correlate of impaired inhibitory mechanisms could be lactate-induced downregulation of GABA activity in the visual cortex. As mentioned, in MA+ lactate levels increase in the occipital cortex during visual stimulation [20] there is emerging evidence that lactate, besides its role as energy substrate, has a concentration-dependent downregulating effect on GABAergic neurotransmission [22].

As other neurophysiological studies, ours has some methodological shortcomings. For instance, the investigators were blinded during off-line analyses of VEP data, as applied in previous studies by independent groups [15, 73], but not to diagnosis during the recording session, although this is probably only a minor risk for bias. As a matter of fact, in a clinical setting it is quasi impossible to totally blind a neurophysiological study. Even in VEP studies that found no abnormalities in migraineurs and were claimed to be blinded to diagnosis [12, 74, 75], blinding was not perfect for various reasons according to the reported methodology: 1) the neurophysiologist knew which set of responses belonged to each of the 6 blocks of averagings [74], which allows a selection bias in favour of low amplitude responses and thus normal habituation [76]; 2) the neurophysiologist was not blinded to check size [74], to which VEP amplitudes are quite sensitive [77]; 3) after each recording, the investigators guessed the correct diagnosis in more than half of subjects [12]; 4) although the investigators were blinded to diagnosis during the first examination, they were not during the 3 subsequent recording sessions [75].

Though we refer herein to habituation as the common feature of responses to any type of repeated sensory stimuli and to its classical definition of “a behavioral response decrement that results from repeated stimulation and that does not involve sensory adaptation/sensory fatigue or motor fatigue” [78, 79], we cannot totally exclude that changes in the level of attention and contrast pattern adaptation may have influenced our results. This is nonetheless unlikely for the following reasons. In previous studies VEP amplitudes after full field stimulation were not significantly influenced by attention and reaction time task conditions [80, 81]. Moreover, the effects of contrast adaptation on the P1 peak are small (peak time shift approx. 3 ms, amplitude unchanged) and require to take place stimulations lasting about 25 min [82, 83], contrasting with a 3 min 20 sec duration of a VEP recording session in our study.

We also are aware that our samples are relatively small and that clinical correlations are retrospective. Further studies are needed to repeat the analysis in a larger clinical sample with various migraine phenotypes and with a longitudinal, prospective follow-up of patients, allowing to record them during attacks as well as at different time points between attacks.

## Conclusions

To summarize, this study shows that the clinical heterogeneity of migraine with aura is reflected in distinct visual evoked potential profiles. Patients with complex neurological auras differ from those with strictly visual auras by an interictal increase of VEP amplitudes suggestive of an underlying genuine persistent visual cortical hyperexcitability. Whether this is related to CSD features, their effect on cortex and thalamus or to a common neurobiological or genetic denominator between CSD and VEP profile determinants remains to be determined.

## Abbreviations

CSD: cortical spreading depression
GWAS: genome-wide association studies
HV: Healthy Volunteer
MA: migraine with pure visual Aura
MA+: migraine with complex aura
MAtot: total group of migraine with aura patients
MO: migraine without aura
MwA: migraine with aura
VEP: visual evoked potential
